# Management of a Pediatric Patient With Langerhans Cell Histiocytosis Using General Anesthesia

**DOI:** 10.7759/cureus.101506

**Published:** 2026-01-14

**Authors:** Bashaer S Abdulhadi, Meshari S Alzahrani

**Affiliations:** 1 Pediatric Dentistry, King Fahad Armed Forces Hospital, Jeddah, SAU

**Keywords:** chemotherapy, full-mouth rehabilitation, langerhan cell, oral health, pediatric oncology

## Abstract

Langerhans cell histiocytosis (LCH) is an uncommon condition that often calls for input from several specialists, especially when the mouth is involved or when treatment affects the child’s immune system. This report outlines the care of a five-year-old female with multisystem disease who developed fever and eye irritation during chemotherapy. Once her blood counts recovered, she received full dental rehabilitation under general anesthesia because of extensive decay and limited cooperation. Close coordination among oncology, dental, pediatric, and eye-care teams supported her recovery and highlighted the value of timely intervention and organized long-term follow-up.

## Introduction

Langerhans cell histiocytosis (LCH) is a rare hematologic disorder characterized by the clonal proliferation of immune cells expressing CD1a and Langerin, driven by mutations in the MAPK and ERK signaling pathways [1]. The discovery of the BRAF V600E mutation in a significant proportion of LCH cases has enhanced the understanding of its neoplastic nature and pathogenesis [1].

LCH can affect individuals of any age, but it occurs most frequently in young children, with most diagnoses made between one and four years of age [2]. The disease may involve a single system or multiple organ systems, including the skin, bones, hematopoietic tissue, and central nervous system [3]. Oral lesions are often among the earliest manifestations, presenting as gingival swelling, pain, alveolar bone loss, or ulcerative lesions that mimic common dental infections [4,3]. Because of these similarities, LCH is often misdiagnosed as periodontal or infectious disease, leading to delays in treatment [4].

The management of LCH has evolved through international consensus recommendations that standardize diagnostic and therapeutic approaches [5]. Children receiving chemotherapy for LCH are at increased risk for oral complications, including mucositis, delayed wound healing, thrombocytopenia, and neutropenia [6,7]. These conditions complicate dental management and increase susceptibility to infection. in this case, the patient developed multiple dental caries as a result of immunosuppression secondary to chemotherapy, rather than direct LCH involvement.Therefore, multidisciplinary coordination between oncology and dental teams is essential to minimize complications and improve patient outcomes [8,9]. Recent reviews have emphasized the importance of combining oncologic and dental management to optimize oral health outcomes in pediatric patients undergoing cancer treatment [9,10]. The present case report describes a child with multisystem LCH who underwent coordinated dental and oncologic care following chemotherapy, highlighting the critical role of interdisciplinary collaboration in improving pediatric oral health and overall treatment success [10].

Recent reviews have emphasized the importance of combining oncologic and dental management to optimize oral health outcomes in pediatric patients undergoing cancer treatment [10,11]. The present case report describes a child with multisystem LCH who underwent coordinated dental and oncologic care following chemotherapy, highlighting the critical role of interdisciplinary collaboration in improving pediatric oral health and overall treatment success [12].

## Case presentation

A five-year-old girl was brought to the clinic after several weeks of recurrent fever, a persistent non-productive cough, and a widespread papular rash. Laboratory studies indicated anemia and elevated inflammatory markers. A biopsy on the skull revealed a lytic lesion. Multisystem LCH was confirmed by histopathology, which revealed proliferation of dendritic cells with positive staining of CD1a and CD207. The patient was initiated on the chemotherapy plan of the use of vinblastine and corticosteroids as per the recommendations of pediatric oncology guidelines. She developed intermittent fevers for approximately three days before admission, which resolved with antibiotic therapy, and had incidents of conjunctival irritation, which complicated her course. There were no indications that she had any problems with her central nervous system or had any issues with her endocrine system either. Pediatric oncologists, ophthalmologists, and general pediatricians coordinated efforts to closely monitor her overall health during the course of treatment.

Six months after the completion of her chemotherapy, follow-up lab findings demonstrated hematologic recovery. Her absolute neutrophil count was 1.5 × 10^9^ per liter, and her platelet count was above 120 × 10^9^ per liter. Due to continuous discomfort and persistent dental caries in the mouth, the patient was referred for a dental examination.

**Figure 1:**
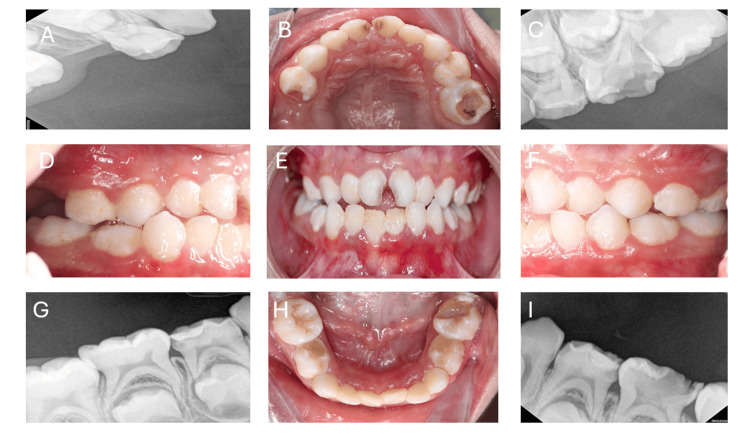
(A-I) Preoperative intraoral radiographs and clinical images illustrating multiple decayed primary teeth. The radiographs highlight the extent of caries in teeth #65, #75, and #84.

Clinical and radiographic examination: Intraoral examination and five periapical radiographs were taken for this patient under general anesthesia before starting the procedure (Figures 1A-1I), which revealed various primary molars with significant tooth decay, extensive plaque buildup on all surfaces of the teeth, and chronic gingival disease caused by inflammation.

In this case, the patient’s oral findings were limited to multiple carious lesions that developed as a consequence of prolonged immunosuppression and poor salivary function secondary to chemotherapy. There was no clinical or radiological evidence of LCH-related osseous or gingival involvement. This distinction is important because oral lesions in LCH can mimic treatment-related changes; however, in this patient, the dental pathology was purely secondary to chemotherapy-induced immune suppression rather than direct disease infiltration. Because the patient was a very young child, uncooperative, and the severity of the disease, we suggested using full mouth dental rehabilitation under general anesthesia for this patient. This method allows for the gradual performance of all restorative and surgical treatments in a single appointment, with minimal patient trauma and risk of infection. Consideration of the child’s oral-health-related quality of life was emphasized, as dental rehabilitation during ongoing chemotherapy can significantly impact comfort and functional recovery.

Pre-anesthetic evaluation included a complete blood count, liver and renal function tests, and radiological assessment to rule out multisystem involvement. The patient had received previous chemotherapy and corticosteroid therapy, which were considered during anesthetic planning. Immunosuppressive status was assessed to minimize perioperative infection risk, and stress-dose steroids were administered to prevent adrenal insufficiency. Airway assessment revealed no obstructive lesions related to LCH involvement. The full-mouth rehabilitation procedure included restoration, pulpotomies, and the placement of stainless-steel crowns on several teeth.

**Figure 2:**
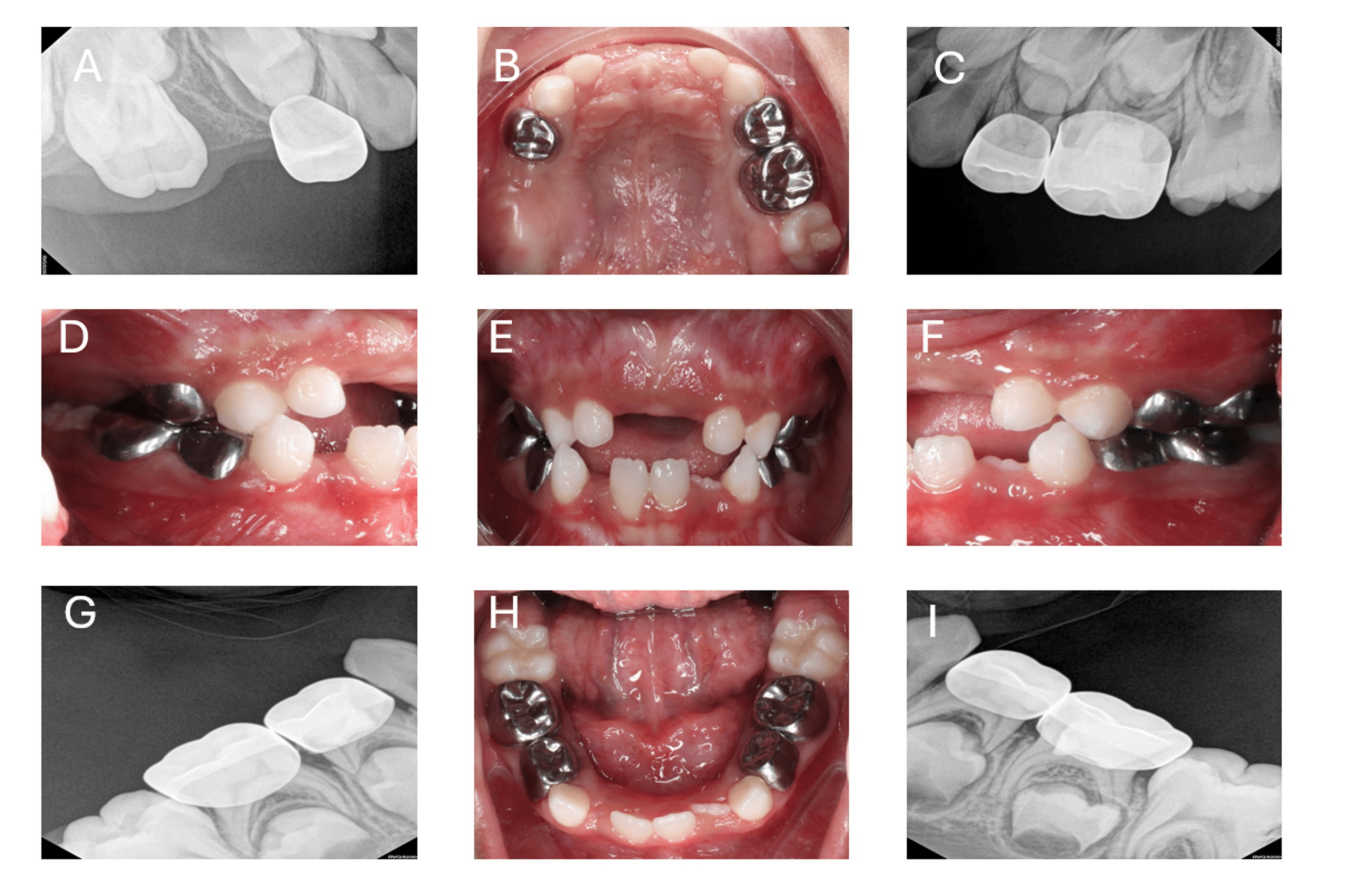
(A-I) Six-month postoperative radiograph and photograph showing successful healing of pulpotomized teeth, excellent soft tissue healing, and intact stainless-steel crown.

During the anesthetic induction and intubation, particular care was taken to avoid excessive neck extension and jaw pressure to prevent trauma to potentially affected bones. General anesthesia was induced using propofol and maintained with sevoflurane in an oxygen-nitrous oxide mixture, with fentanyl for analgesia and atracurium as a muscle relaxant. Pain management included multimodal analgesia with paracetamol and low-dose opioids, ensuring adequate control while avoiding excessive sedation. The patient had an unremarkable recovery from anesthesia and was discharged home the day of surgery with postoperative care. The oral cavities were noted to be entirely healed at a follow-up visit one month later. In six months follow-up, radiographic X-rays and intraoral photographs (Figures 2A-2I) show complete healing of the gingiva and good oral hygiene normal periapical area of all pulpotomized teeth and a stainless-steel crown.

The patient maintained a stable clinical condition and did not experience a resurgence of LCH in the next six months.

## Discussion

LCH is a heterogeneous disorder that can present as a single-lesion or multisystem disease, both of which may be life-threatening. The pathophysiology of LCH involves mutations in hematopoietic progenitor cells that activate the MAPK and ERK signaling pathways, leading to abnormal proliferation, survival, and differentiation of myeloid lineage cells [1]. These clonal mutations result in the accumulation of Langerhans-like dendritic cells that infiltrate tissues and drive inflammation, fibrosis, and tissue damage [1].

Children with multisystem LCH frequently require systemic chemotherapy as a cornerstone of management. However, treatment-related complications can make comprehensive care challenging, particularly when addressing oral or dental manifestations of the disease [3]. Dental professionals must balance the side effects of chemotherapy - such as mucositis, thrombocytopenia, and neutropenia - with the need to control oral infection and maintain function [6,7].

Oral lesions in children with LCH often mimic common dental pathologies, such as periodontitis or osteomyelitis, which can delay diagnosis and treatment [4]. This overlap emphasizes the importance of interdisciplinary collaboration between dental and medical teams for accurate diagnosis and early intervention [5]. Dental care in these patients requires careful planning to minimize bleeding risks and infection during chemotherapy-induced immunosuppression [8].

General anesthesia may be indicated for children with LCH due to behavioral, developmental, or systemic considerations that limit cooperation during complex dental procedures. In the present case, general anesthesia was chosen to ensure procedural safety and minimize stress for the pediatric patient. Multidisciplinary coordination between dental, oncologic, and anesthetic teams is essential for successful outcomes, as emphasized in pediatric oncology protocols [10]. Post-chemotherapy care should include regular oral evaluations, imaging, and close monitoring to detect potential disease recurrence or complications [3].

A multidisciplinary approach - including oncologists, radiologists, pathologists, and pediatric dentists - is essential for optimizing long-term outcomes in LCH management [11]. Such coordination facilitates early recognition of oral lesions, effective infection control, and the prevention of systemic complications. Ultimately, integrated oncologic and dental care contributes to better patient comfort, enhanced healing, and a reduced risk of treatment-related morbidity [12].

Recommendation

Table 1 presents the recommendations for treatment protocols for patients with LCH.

**Table 1:** Recommendations for the treatment protocols for patients with LCH. LCH: Langerhans cell histiocytosis

Recommendation	Key Action/Focus	Rationale/Benefit
Early Introduction to Dental Care	Introduce children to dental services shortly after diagnosis	Helps identify existing oral issues and informs the development of a preventive plan tailored to the child's medical status [4,8]
Regular and Predictable Follow-up	Visits scheduled every three to six months	Allows for close monitoring, reinforcement of hygiene practices, prompt management of emerging concerns, and strengthens the child’s comfort with the dental environment [8,11]
Close Coordination with Medical Specialists	Dental team maintains regular communication with oncology and pediatric care providers	Ensures that dental procedures are scheduled safely by sharing laboratory results, treatment updates, and clinical concerns [5,10]
Emphasis on Preventive Strategies	Application of fluoride varnish, dietary counseling, plaque-control instruction, and early intervention for caries-prone teeth	Reduces the likelihood of major dental treatment in the future [8,11]
Clear Emergency Protocols	Families receive guidance on managing dental pain, trauma, or signs of infection	Helps avoid unnecessary hospital visits and ensures prompt treatment when urgent issues arise [5,7]

## Conclusions

Caring for a child with LCH calls for the combined efforts of several healthcare providers, each bringing a different lens to the child’s overall health. In this case, steady coordination and careful timing allowed the team to manage the patient’s medical needs while restoring her oral health. Once her blood counts recovered, completing her dental treatment became both safe and beneficial. Restoring her teeth after chemotherapy not only reduced the risk of future infections but also supported her broader recovery, highlighting how essential coordinated dental care is for children living with LCH.
